# Supplementary material for: Observation of a bilayer superfluid with interlayer coherence
Source: Nat Commun. 2025 Aug 5;16:7201. doi: 10.1038/s41467-025-62277-w (PMC12325720; doi:10.1038/s41467-025-62277-w)
Supplement: Supplementary file 1 — Supplementary Information [file 41467_2025_62277_MOESM1_ESM.pdf]

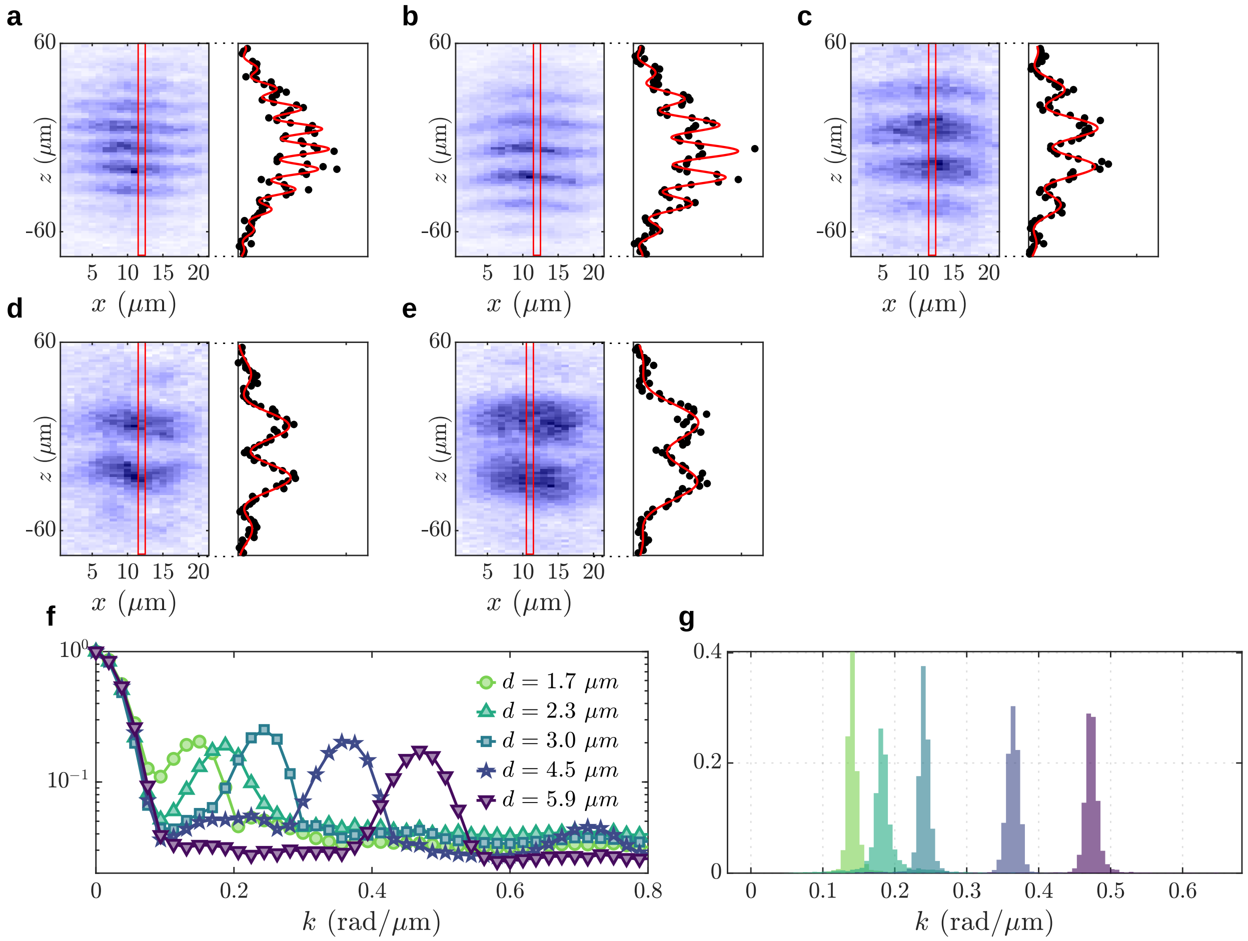

Supplementary Fig. 1. **Matter-wave interference patterns and their spectrum. a-e**, Matter-wave interference images at $d = 5.9\mu m$ (a), $d = 4.5\mu m$ (b), $d = 3.0\mu m$ (c), $d = 2.3\mu m$ (d), $d = 1.7\mu m$ (e). The panels on the right show the density distribution along the central column, in arbitrary units, together with a fit with model (M4). **f,** Normalized spectra along the z directions, of the experimental images, plotted for different well separations d. The low-momentum peak comes from the envelope of the 2D cloud following the TOF expansion, and their nearly complete overlap demonstrates the same vertical trapping parameter; the expansion along z is that of a Gaussian ground-state wavepacket for quasi-2D regime [63]. **g,** Distributions of fitted values of the interference fringe wavelengths. The peak locations are unchanged from panel **f**, while the peak is narrowed by a non-linear least squares fitting routine.

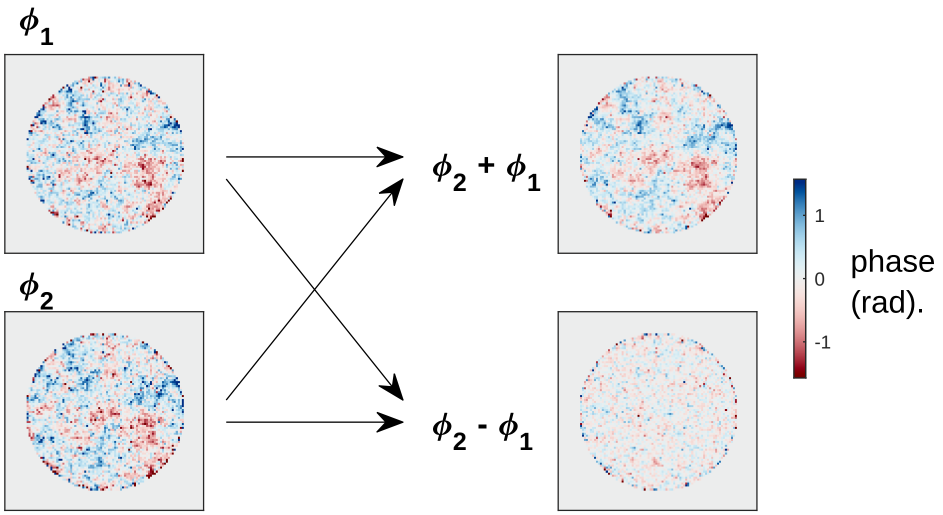

Supplementary Fig. 2. **Analysis of numerical simulation**. From the simulated phase fields ϕ_1 and ϕ_2, we obtain symmetric and antisymmetric modes from which the phase correlation functions are computed.

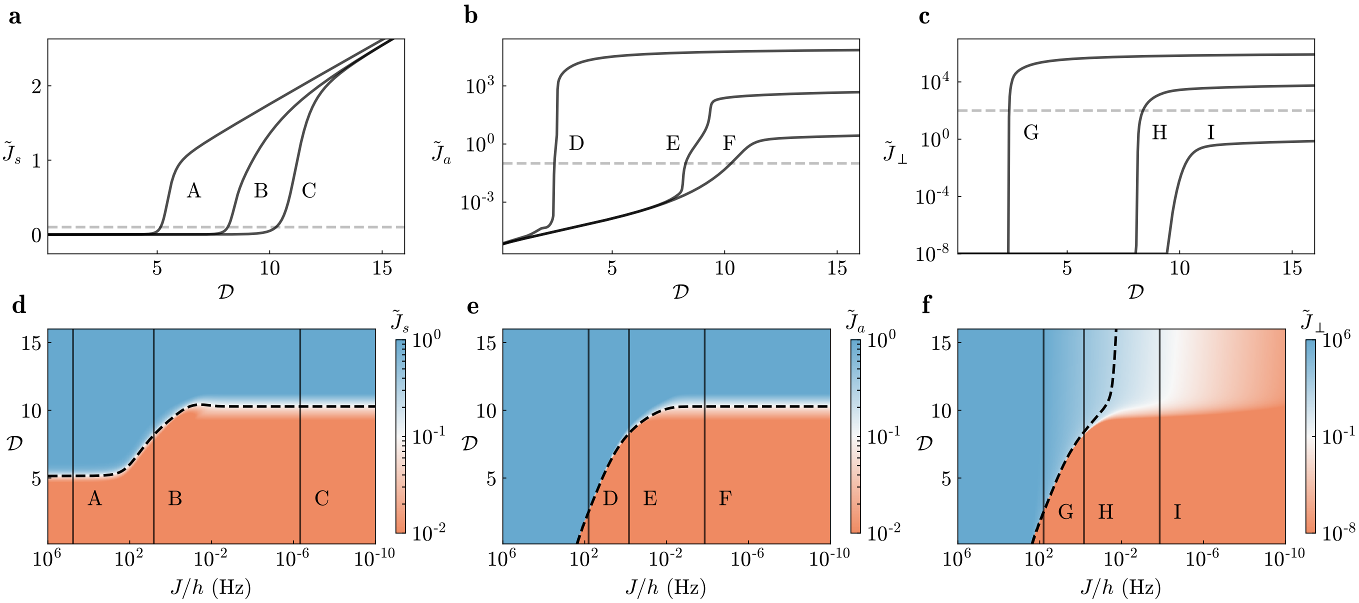

Supplementary Fig. 3. **Renormalization-group results for the phase stiffness.** The Renormalization-Group theory analysis was carried out using the numerical integration of Eqs. (M8), with $T' = 208$ Hz, $A_{1,0} = 0.345T'/\pi$ and $J_{s,0} = J_{a,0} = \frac{\mathcal{D}}{5.5}T'/\pi$, $A_{a,0} = A_{s,0} = A_{1,0}$, constants $\alpha_2 = 5.7$, $\alpha_3 = 0.1$ and $\Delta l = 7$. **a,b** The values of the dimensionless phase stiffnesses $\tilde{J}_{s,a} = \pi J_{a,s}/T'$ at the end of integration at different coupling strengths. Dashed lines denote the 10^{-1} dimensionless cutoff chosen to distinguish between the superfluid and disordered phases. **c** Value of the dimensionless interlayer coupling strength $\tilde{J}_\perp = \pi J_\perp/T'$, at the end of RG integration, dashed line denotes $\tilde{J}_\perp = 10^2$. **d,e** The values of $\tilde{J}_s$ and $\tilde{J}_a$ after RG integration. The contour lines (dashed) are where the values of the stiffnesses cross 10^{-1} which we identify as the critical point for a given value of J. **f** Value of $\tilde{J}_\perp$ at the end of RG integration. In the DLSF and ASF phases $\tilde{J}_\perp \gg 1$ under RG, the dashed contour shows where $\tilde{J}_\perp$ crosses 10^2.

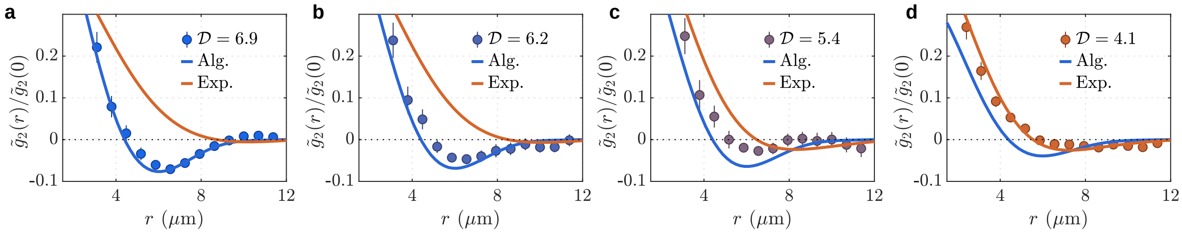

Supplementary Fig. 4. **Density noise correlation functions across the critical point.** Radially averaged noise correlation functions $\tilde{g}_2(r)$ for the $d = 1.7\,\mu$m dataset also shown in Fig. 3c. The best fits from Eq. (M5), corresponding to algebraically (exponentially) decaying form of the common phase correlations are shown in blue (orange), where the shapes depend on the value of the algebraic (exponential) decay parameters (see Methods). Data shown for phase-space densities are **a**, $\mathcal{D} = 6.9$, **b**, $\mathcal{D} = 6.2$, **c**, $\mathcal{D} = 5.4$, **d**, and $\mathcal{D} = 4.1$.
